# Investigation of a Suspected Autochthonous Cholera Case in Brazil After an 18-Year Absence of Reported Cases

**DOI:** 10.1590/0037-8682-0558-2025

**Published:** 2026-06-15

**Authors:** Maria Goreth Matos de Andrade Barberino, Ana Verena Almeida Mendes, Gabriela Noronha Marques, Camila Araújo de Lorenzo Barcia, Marcela Almeida Muhana, Aline Macedo Carvalho Freitas, Mariana Leal de Souza Mercês, Ênio Silva Soares, Lázaro José Rodrigues de Souza, Arabela Leal e Silva de Mello, Felicidade Mota Pereira, André Felipe das Mercês Santos, Dália dos Prazeres Rodrigues, Guilherme de Sousa Ribeiro, Cristiane Wanderley Cardoso

**Affiliations:** 1 Hospital São Rafael, Salvador, BA, Brasil.; 2 Instituto D’OR de Pesquisa e Ensino (IDOR), Salvador, BA, Brasil.; 3 Escola Bahiana de Medicina e Saúde Pública, Salvador, BA, Brasil.; 4 Secretaria Municipal de Saúde de Salvador, Centro de Informações Estratégicas em Vigilância em Saúde, Salvador, BA, Brasil.; 5 Secretaria de Saúde do Estado da Bahia, Laboratório Central do Estado da Bahia, Salvador, BA, Brasil.; 6 Fundação Oswaldo Cruz, Instituto Oswaldo Cruz, Laboratório de Enterobactérias, Rio de Janeiro, RJ, Brasil.; 7 Universidade Federal da Bahia, Escola de Medicina da Bahia, Salvador, BA, Brasil.; 8 Fundação Oswaldo Cruz, Instituto Gonçalo Moniz, Salvador, BA, Brasil.

**Keywords:** Cholera, Vibrio cholerae, Epidemiological surveillance

## Abstract

Cholera has caused multiple pandemics and remains a significant global public health threat. After 18 years without autochthonous transmission in Brazil, a 60-year-old man with no travel history presented with abdominal discomfort and acute watery diarrhea. Laboratory testing detected *Vibrio cholerae* O1 Ogawa, prompting classification as a suspected cholera case. However, genomic analysis showed that the strain lacked the toxigenic virulence factor required to cause cholera, leading to its reclassification as a non-cholera case. This investigation highlights the importance of clinical suspicion, advanced laboratory diagnostics, and continuous epidemiological surveillance for accurate case identification and an appropriate public health response.

## INTRODUCTION

Cholera has caused millions of deaths across seven pandemics and remains a major public health threat. Between 1 January and 12 December 2024, more than 751,400 cholera cases and over 5,200 deaths were reported globally across 33 countries, making cholera outbreaks one of the most pressing health emergencies^1^. *Vibrio cholerae* is a free-living aquatic bacterium that can cause cholera in humans, an acute diarrheal disease resulting from toxins produced by strains carrying the *ctxAB* gene. These strains primarily belong to the O1 and O139 serogroups, although not all strains within these serogroups carry the *ctxAB* gene. Non-toxigenic strains may cause mild diarrhea; however, these infections are not typically classified as cholera^2^. 

In Brazil, no autochthonous cholera cases have been reported since 2006, when cases were recorded in Pernambuco, a northeastern state, although occasional imported cases from Africa and Asia have been documented^3^. However, in 2024, a suspected autochthonous case was identified in the municipality of Salvador, Bahia. The patient met the Brazilian Ministry of Health criteria for suspected cholera (individuals ≥5 years of age from non-outbreak areas presenting with abrupt, profuse watery diarrhea)^3^ and had *V. cholerae* O1 Ogawa detected in stool. 

A systematic search of multiple databases (Cochrane Library, LILACS, MEDLINE, PubMed, PubMed Central, and SciELO), updated through April 2026, identified only a single correspondence reporting the potential re-emergence of cholera in Brazil^4^. This epidemiological surveillance investigation describes a probable autochthonous cholera case in Brazil after an 18-year absence. The study was approved by the Ethics Committee of the Instituto D’Or de Pesquisa e Ensino (CAAE # 82855024.2.0000.0048) and conducted in accordance with Good Clinical Practice guidelines.

## CASE REPORT

Between March 16 and 19, 2024, a 60-year-old mixed-race man with no comorbidities other than arterial hypertension developed abdominal discomfort and acute watery diarrhea (five to six non-bloody stools per day, without vomiting). He reported no recent travel to cholera-affected areas and no known contact with suspected or confirmed cases. On March 25, he developed a fever and, the following day, sought care at a private hospital. Laboratory parameters were within normal limits, except for an elevated C-reactive protein (CRP) level (5.26 mg/L; reference value: <1.0 mg/L). The dengue NS1 rapid test was negative. He received symptomatic treatment and was discharged with a prescription for medication for abdominal pain. No stool samples were collected during this visit. 

On March 28, he felt unwell at work, with persistent abdominal discomfort and watery diarrhea accompanied by dizziness. His blood pressure was noted to be low, prompting a visit to the emergency department of another private hospital. Laboratory tests showed increased inflammatory markers, including leukocytosis (18,190 cells/μL) and elevated C-reactive protein (289.7 mg/L) (Table 1). An abdominal computed tomography (CT) scan revealed thickening of the intestinal loops involving the rectum and the ascending, transverse, and descending colon, along with mesenteric fat stranding and hydroaerial content within the loops, suggesting inflammatory involvement. Because of persistent symptoms and laboratory abnormalities, the patient was admitted and started on intravenous hydration, ceftriaxone, and metronidazole.

**TABLE 1:** Clinical and laboratory findings of a suspected cholera case during hospital admission, Salvador, Brazil, 2024.

Clinical and laboratory evaluation	Findings according to the date of hospitalization in 2024						
	28 mar	29 mar	30 mar	31 mar	1 Apr	2 Apr	3 Apr
Temperature (°C) (Min-Max)	37.9 (36.4-37.9)	37.5 (35.2-37.5)	37.0 (35.4-37.0)	37.2 (36.1-37.2)	ND	37.1 (36.8-37.1)	37.0 (35.2-37.0)
Non-bloody watery diarrhea	Yes	Yes	Yes	Yes	Yes	No	No
Hemoglobin (g/dl)^1^	13.7	13.5	ND	14.3	13.3	13.3	13.8
Hematocrit (%)^1^	41.2	41.9	ND	44.2	41.6	40.4	43
Lymphocytes (10^3^/mm³)^1^	0.88	1.09	ND	1.12	1.41	1.43	1.73
Platelets (10^3^/mm³)^1^	222	228	ND	275	276	293	304
Leukocytes (10^3^/mm³)^1^	15.38	18.19	ND	8.44	7.24	6.94	7.38
Sodium (mmol/L)^2^	132	137	ND	136	136	132	135
Potassium (mmol/L)^2^	4.0	3.6	ND	3.7	4.0	4.5	4.8
Magnesium^1^ (mmol/L)^2^	ND	ND	ND	0.8	0.8	0.8	0.9
Serum Phosphorus (mg/dl)^2^	ND	ND	ND	ND	4.0	3.5	3.7
C-reactive protein (mg/L)^2^	286.8	289.7	ND	133.8	78.6	55.7	40
Glucose (mg/dl)^2^	ND	ND	ND	89	ND	ND	86
Lactate (mg/dl)^2^	10	ND	ND	ND	7.0	12	11
Total Bilirubin (mg/dl)^2^	1.3	ND	ND	ND	0.4	0.4	0.4
Creatinine (mg/dl) ^3^	1.28	0.84	ND	0.92	0.79	0.69	0.82
Urea (mg/dl)^3^	25	19	ND	17	22	20	19

**ND:** No Data. Reference values (Medscape: https://emedicine.medscape.com): ^1^ **Hematology (Blood count):** Hemoglobin: 14-18 g/dL; Hematocrit:42-50%; Lymphocytes:1000-4000/mm^3^; Platelets: 150,000- 450,000/mm³); Leukocytes: 5,000 to 10,000/mm³).^2^ **Electrolytes and Metabolic:** Sodium: 136-145 mmol/L; Potassium: 3.5-5 mmol/L; Magnesium: 0.65-1.05 mmol/L; Serum Phosphorus: 3.0-4.5 mg/dL; C-reactive protein: 1.0 mg/dL; Glucose: 74-106 mg/dL; Lactate: 4.5-9 mg/dL; Bilirubin: 0.3-1.0 mg/dL. ^3^ **Renal function analysis:** Creatinine: males: 0.6-1.2 mg/dL; Urea: 10-20 mg/dL.

Stool samples were negative for *C. difficile* toxins A and B (fluorescence immunoassay)^5^ and rotavirus antigens (chromatography and enzyme immunoassay)^6^. Stool culture yielded yellow-translucent colonies consistent with *V. cholerae*, confirmed by Matrix-Assisted Laser Desorption/Ionization Time-of-Flight Mass Spectrometry (MALDI-TOF MS) (bioMérieux, Marcy l’Etoile, France) with 99.9% confidence. On March 30, following detection of *V. cholerae*, antimicrobial therapy was adjusted to a single 300 mg oral dose of doxycycline, after which abdominal pain improved; however, watery, non-bloody diarrhea (5-6 episodes/day, without vomiting) persisted until April 1, when it resolved. 

On the same day that the hospital reported a suspected autochthonous cholera case to the Salvador surveillance office, the Center for Strategic Information in Health Surveillance of Salvador (CIEVS-SSA) initiated an epidemiological investigation and response. This included the collection of sociodemographic and clinical data through medical record review and standardized interviews, contact tracing, and notification to state surveillance authorities (including the State Central Laboratory, LACEN-BA), the Brazilian Ministry of Health, and the Pan American Health Organization/World Health Organization. On April 19, 2024, the Brazilian Ministry of Health issued a technical note reporting the case and recommending strengthened epidemiological surveillance for acute diarrheal diseases and cholera^7^.

During the contact investigation, stool samples were collected from healthcare professionals who assisted the patient at the two hospitals he attended (hospital #1: 6 contacts; hospital #2: 57 contacts) and from seven of the patient’s friends and family members. All contacts were asymptomatic and tested negative, except for the patient’s 49-year-old wife, whose stool culture also yielded *V. cholerae*. She remained asymptomatic and did not receive prophylactic antibiotics, in accordance with Ministry of Health recommendations^3^. Food samples (fish from the market frequented by the patient) and household water samples from the patient’s home and the homes of his contacts were also collected; all tested negative for *V. cholerae*. 

Subsequent analysis at the national reference laboratory (FIOCRUZ, Ministry of Health) confirmed the presence of *V. cholerae* in the stool samples of the patient and his wife using a multiplex polymerase chain reaction (PCR) assay. The patient’s isolate belonged to serogroup O1, but not to the El Tor lineage. PCR screening for toxin-related genes detected *omp*W, *amp*U, *tox*, *tcp*, and *ace*, whereas *ctx*AB*, rfb*O1, *zot*, *rtx*, and *hly*A were absent. In contrast, his wife’s isolate belonged to the non-O1 lineage.

**FIGURE 1:**
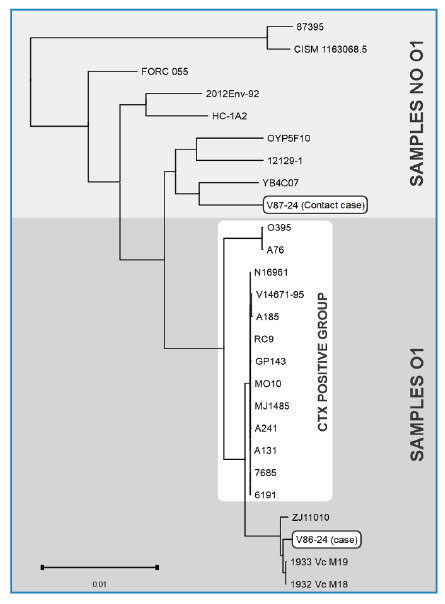
Dendrogram with strains of *Vibrio cholerae* O1 and non-O1, where two groups stand out (*V. cholerae* O1 and *V. cholerae* non-O1).

Whole-genome sequencing (WGS) and single-nucleotide polymorphism (SNP) analysis were performed to compare the genetic similarities among the *V. cholerae* strains from the suspected case, his wife, and reference strains (Table 2; Figure 1). The strains from the patient and his wife differed by > 40,000 SNPs. Because differences greater than 10,000 SNPs typically indicate deep evolutionary divergence, this large genetic distance suggests that the strains are phylogenetically unrelated and not epidemiologically linked, with no evidence of direct transmission of *V. cholerae* between them. Comparisons with genomes of isolates collected in different years and countries showed that the patient’s strain was most similar to O1 strains from Mexico and China. 

**TABLE 2:** Genomic distances based on SNPs between the reference (case strain), contact suspected case (V87/24), and the remaining isolates.

Sample	Year	Country	SNP´s variations	Serogroup
Vc_M18	2014	Mexico	3846	O1
Vc_M19	2004	Mexico	3927	
ZJ11010	2011	China	5895	
N16961	1982	Bangladesh	14748	
RC9	1985	Kenya	14775	
MO10	1992	India	14963	
GP143	1978	Bahrein	14993	
7685	2009	Kenya	14996	
6191	2005	Kenya	15025	
MJ1485	1994	Bangladesh	15059	
A131	1989	India	15088	
V14671-95	1995	Brazil	15231	
A241	1989	Vietnam	15252	
A185	1992	Colombia	15320	
A76	1982	Bangladesh	28057	
O395	1965	India	28184	
V87-24	2024	Brazil	47114	Not O1
YB4C07	2009	USA	49442	
OYP5F10	2009	USA	50000	
12129-1	1985	Australia	50177	
2012Env-92	2012	Haiti	59313	
HC-1A2	2010	Haiti	60166	
FORC_055	2010	South Korea	61216	
CISM_1163068.5	2012	Mozambique	116360	
87395	1983	Mexico	116642	

**SNPs** (single-nucleotide polymorphisms) are single-base variations at specific positions in the genome between isolates; >40,000 SNPs indicates substantial genetic divergence, consistent with unrelated strains and no recent epidemiological linkage.

## DISCUSSION

In this case report, we describe an acute diarrheal illness in which laboratory testing initially identified *V. cholerae* serogroup O1 Ogawa, raising suspicion of an autochthonous cholera case. However, further molecular analyses revealed that the O1 strain did not belong to the typically toxigenic El Tor lineage. Consequently, in December 2025, the Brazilian Ministry of Health issued a technical note reclassifying the case as non-cholera^8^. Although nontoxigenic *V. cholerae* does not cause cholera, it remains epidemiologically relevant because it can participate in horizontal gene transfer and recombination, potentially contributing to the emergence of new toxigenic strains. Furthermore, nontoxigenic strains can cause other types of gastrointestinal vibriosis^9^.

Evidence from Asia, particularly Bangladesh, indicates that nontoxigenic and non-O1/non-O139 *V. cholerae* strains can cause cholera-like diarrheal outbreaks, including large epidemics that are clinically indistinguishable from cholera. Notably, such outbreaks may occur alongside toxigenic O1 transmission, posing challenges for surveillance, laboratory confirmation, and outbreak attribution^10^ ^,^ ^11^. These findings underscore the epidemiological relevance of the nontoxigenic O1 strain identified in our patient and reinforce the need for careful molecular characterization and continued monitoring, particularly because cholera is a notifiable disease in Brazil^8^ and elsewhere.

This suspected cholera case, which required care at two different hospitals, highlights the importance of considering cholera in the differential diagnosis of patients presenting with acute diarrhea. Although ultimately a false alarm, the event provided an opportunity to evaluate the surveillance system’s ability to detect, investigate, and respond to potential public health threats. From an epidemiological perspective, factors such as climate change, complex humanitarian crises, poor sanitation, and limited preventive measures-including vaccines-pose ongoing risks^12^. Together, these challenges reinforce the need for sustained investment in surveillance, prevention, and preparedness capacity.

## Data Availability

Research data is only available upon request.
